# Computer programmers show distinct, expertise-dependent brain responses to violations in form and meaning when reading code

**DOI:** 10.1038/s41598-024-56090-6

**Published:** 2024-03-05

**Authors:** Chu-Hsuan Kuo, Chantel S. Prat

**Affiliations:** 1 Department of Psychology, University of Washington, Seattle, WA USA; 2 Institute for Learning and Brain Sciences, University of Washington, Seattle, WA USA

**Keywords:** Language, Computer science

## Abstract

As computer programming becomes more central to the workforce, the need for better models of how it is effectively learned has become more apparent. The current study addressed this gap by recording electrophysiological brain responses as 62 Python programmers with varying skill levels read lines of code with manipulations of form (syntax) and meaning (semantics). At the group level, results showed that manipulations of form resulted in P600 effects, with syntactically invalid code generating more positive deflections in the 500–800 ms range than syntactically valid code. Meaning manipulations resulted in N400 effects, with semantically implausible code generating more negative deflections in the 300–500 ms range than semantically plausible code. Greater Python expertise within the group was associated with greater sensitivity to violations in form. These results support the notion that skilled programming, like skilled natural language learning, is associated with the incorporation of rule-based knowledge into online comprehension processes. Conversely, programmers at all skill levels showed neural sensitivity to meaning manipulations, suggesting that reliance on pre-existing semantic relationships facilitates code comprehension across skill levels.

## Introduction

As computer programming, or coding, has moved from being a niche skill to one that is broadly valued in STEM fields and the workforce, the need for improved models of what underlies skilled programming has become more apparent. Fortunately, an increasing amount of research has been devoted to understanding the cognitive and neural bases of computer literacy^1–3^. This body of work is critical and timely, as converging evidence points to a mismatch between the canonical ways that computer programming is taught, and the way it is best learned^4^. In fact, statistics suggest that as many as 50% of students who enroll in Intro Programming courses worldwide drop them before completion^5^.

In their formative paper “The language of programming: A cognitive perspective,” Fedorenko and colleagues^6^ outlined many theoretical parallels between the cognitive bases of programming languages and natural languages. In it, they argue that similarities in the cognitive underpinnings of code and language comprehension and production offer opportunities to “reconceptualize the way [computer science] is taught, especially in early childhood, when children are learning to read and write.” The use of reading and writing as educational comparisons for learning to code is particularly compelling. Unlike the spoken and signed aspects of *native* language acquisition, which occur largely implicitly through reinforcement learning mechanisms, reading and writing are skills that must interface with pre-existing linguistic knowledge, and they are typically explicitly taught in classroom environments. We believe that this is an important distinction to make when it comes to learning programming languages. In the current experiment, when considering natural language learning as a model for understanding how programming languages are learned, our focus is on the explicitly taught and learned aspects of natural language. Specifically, we argue that learning to code might resemble instructed (as opposed to immersed) second language (L2) learning.

Recent research investigating the neural and cognitive underpinnings of learning programming languages has adopted two main approaches: (1) investigating the predictors of facile code learning (e.g., those comparing the predictive utility of numeracy and literacy)^3,4,7^; and (2) investigating the neural underpinnings of code comprehension^1,2,8^. Evidence gleaned from both approaches suggests that explicit L2 learning environments may be useful models for understanding how programming languages are learned. For example, in their investigation of the predictors of learning to program in Python, Prat and colleagues^3^ found that the Modern Language Aptitude Test^9^, developed to detect variability in the ability to learn an L2 in adulthood, explained approximately 30% of the variance in both learning rate and ultimate programming skill obtained. In an fMRI experiment exploring the neural basis of code comprehension, Floyd and collaborators^1^ found that the similarities in neural responses when native English speakers read either code or English were higher in expert coders than in novices. A similar pattern was observed in a series of meta-analyses of bilingual language representation conducted by Sebastian, Laird, and Kiran^10^, with higher proficiency bilinguals showing greater similarity in patterns of activation between their L2 and native languages than did bilinguals with low or moderate proficiency levels.

In the current experiment, we aim to extend and refine this research by exploring the *online* neural responses of programmers at varying levels of expertise as they comprehend lines of code incrementally, in real time. To do so, we employed two electrophysiological indices, the N400 and P600, which have been used extensively to study online language comprehension processes in both native language speakers and L2 learners. Though the precise neurocomputational nature of these components remains debated^11^, the N400—a negative deflection in the event-related potential (ERP) that peaks approximately 400 ms after stimulus onset—has been shown to be sensitive to factors that influence the accessibility of a stimulus’s meaning, or semantics, from memory (e.g., word frequency, semantic congruity, and semantic predictability)^12^. Meanwhile, the P600—a positive deflection that peaks approximately 600 ms after stimulus onset—has been shown to be sensitive to structural violations, or syntax (e.g., subject-verb agreement violations or word order violations)^13^. Although prevalent in the neurolinguistics literature, these ERP components have also been observed when participants engage in meaning-making using other familiar symbolic systems, such as mathematics^14^.

Critically for our questions of interest, these ERP components have been shown to index expertise^15,16^ and individual differences more broadly^14,17^ by measuring the real-time brain processes that reflect our ability to make sense of incremental stimuli at the local (e.g., word) and broader structural (e.g., sentence) levels. Specifically, converging evidence from both longitudinal^15^ and cross-sectional^16^ studies of L2 learning suggest that as individuals progress from novice to advanced proficiency levels in their L2s, sensitivity to violations in form (or syntax) begin to look more like native language violations, increasingly evoking the P600 response. In their review of this work, McLaughlin and colleagues^15^ describe these findings as evidence of “grammaticalization,” or “the instantiation of rule-based knowledge into the learner’s real-time language processing systems” (p. 126)^15^. With respect to skilled L2 comprehension, they propose that “learners must somehow separate the linguistic input into those aspects related to meaning and those related to form.”

In the current study, we explore the extent to which these real-time indicators of sensitivity to form and meaning might be applied to our understanding of how learners of different skill levels comprehend lines of code. We chose Python as the programming language of choice because it has quickly risen to be amongst the most popular programming languages, with a development philosophy centered on the idea of being “reader friendly.” Additionally, an increasing amount of contemporary research on code comprehension has focused on Python^2,3,18^, though some research suggests that the central predictors of learning to code are common across programming languages^18^.

**Figure 1:**
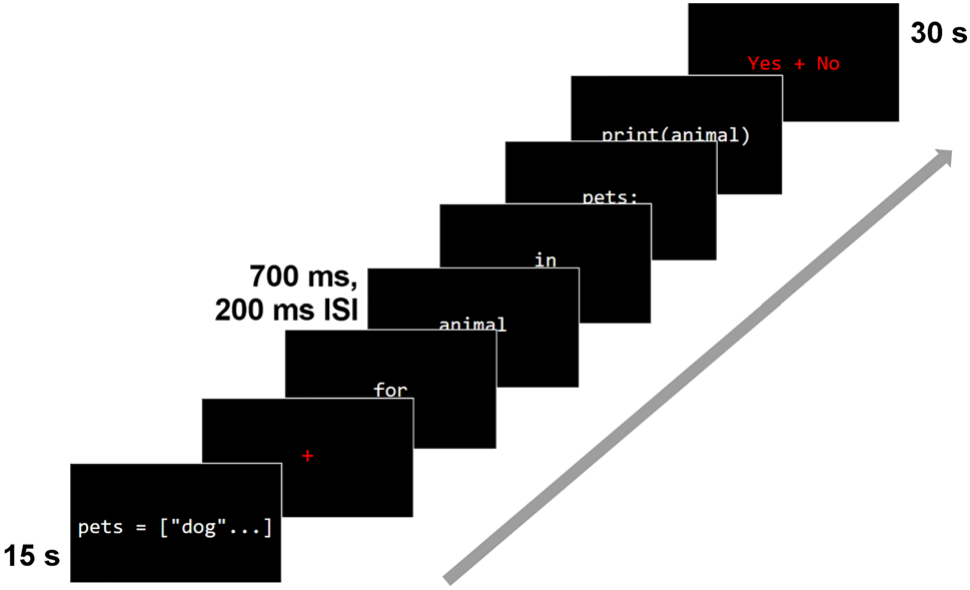
Schematic depiction of a single trial. Following the presentation of the relevant global variable (15 s) for the upcoming trial, the line of code appeared incrementally on an item-by-item basis (700 ms, 200 ms ISI), with “item” defined as the elements of code that appeared between spaces. Participants made their acceptability judgment by pressing a button corresponding to Yes/No after the line of code finished presenting (30 s).

To measure Python code comprehension, we recorded ERPs from 45 programmers of varying skill levels, assessed using an independent programming task (see "Methods"), as they read lines of code presented incrementally (Fig. 1). Each trial began with the presentation of a global variable on the screen for 15 s. Afterward, a single line of code was presented incrementally, on an item-by-item basis. Items were defined as the parts of code that appeared between spaces. Participants were asked to read the code for comprehension and make a behavioral response about its “acceptability” (a term that was left intentionally vague to prevent biasing attention toward syntactic versus semantic aspects of the code) afterward. Participants read 160 lines of Python in total, consisting of 40 trials of each of the following conditions: (1) well-formed lines of code, (2) code with syntactic violations, (3) code with semantic implausibility, and (4) code with both semantic implausibility and syntactic violations. See the "Methods" section for a more detailed description of the stimuli.

This research was designed to answer two questions: (1) Do the P600 and N400 ERP components index sensitivity to form and meaning when programmers comprehend lines of code incrementally? and (2) Does sensitivity to form change as a function of programming proficiency?

Using L2 comprehension as a model, we predicted that manipulations of semantic plausibility would elicit N400 responses, while violations of syntactic validity would result in P600 responses. If evidence for increasing “grammaticalization” occurs when people learn a programming language, we would predict an interaction between Python expertise and the types of neural responses observed to our manipulations of well-formedness. Taken together, this study allows us to examine how coders of varying proficiency levels incrementally incorporate information about form and meaning into their mental representations of lines of code.

## Results

### Behavioral code acceptability judgments

**Figure 2:**
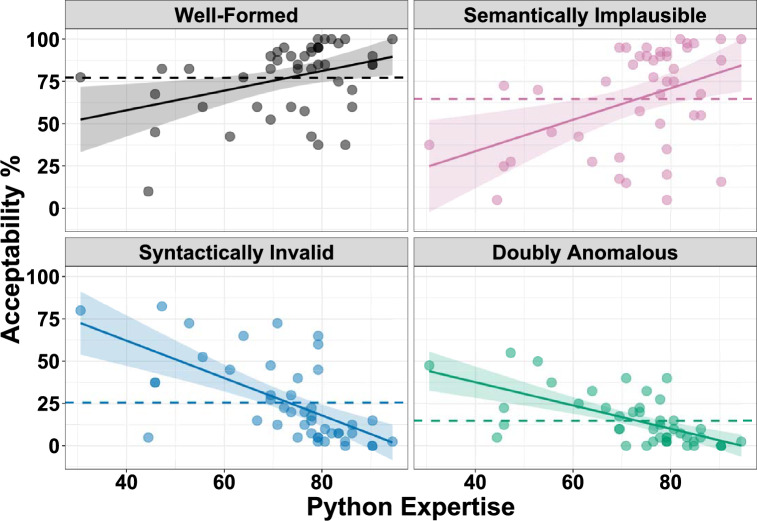
Acceptability judgments (%) for the four code conditions as a function of Python expertise (% on a Python knowledge test). The shaded region around the trend lines indicate the 95% confidence interval. The horizontal dashed lines indicate the mean code acceptability rate for each condition.

The overall model predicting acceptability judgments has a total explanatory power of 73.70%, in which the fixed effects explain 66.30% of the variance. The model’s intercept is at 45.59 (*SE* = 1.96, 95% CI [41.75, 49.25], *t*(43) = 23.29, *p* < 0.001). Within this model, the effects of semantic plausibility and syntactic validity were both significant in predicting acceptability judgments. Specifically, participants judged well-formed code with higher acceptability rates than conditions where semantic implausibility (*b* = 11.66, *SE* = 2.69, 95% CI [6.39, 16.92], *t*(130) = 4.34, *p* < 0.001) or syntactic violations (*b* = 50.67, *SE* = 2.69, 95% CI [45.51, 55.94], *t*(130) = 18.87, *p* < 0.001) occurred (Fig. 2). The effect of syntactic validity was modified by an interaction with Python expertise, with higher Python expertise being associated with lower acceptability judgments to code containing syntactic violations (*b* = 22.81, *SE* = 2.69, 95% CI [17.53, 28.09], *t*(130) = 8.47, *p* < 0.001). On the contrary, the interaction between semantic plausibility and Python expertise approached significance in the opposite direction, with higher Python expertise being associated with higher acceptability judgments to code containing semantic implausibility (*b* = -5.30, *SE* = 2.70, 95% CI [− 10.58, − 0.03], *t*(130) = − 1.97, *p* = 0.051). The other fixed effects included in the model did not reach significance. Full model results for all fixed effects and random components are reported in Supplementary Tables S1–S3.

### Group-level ERP analyses

**Figure 3:**
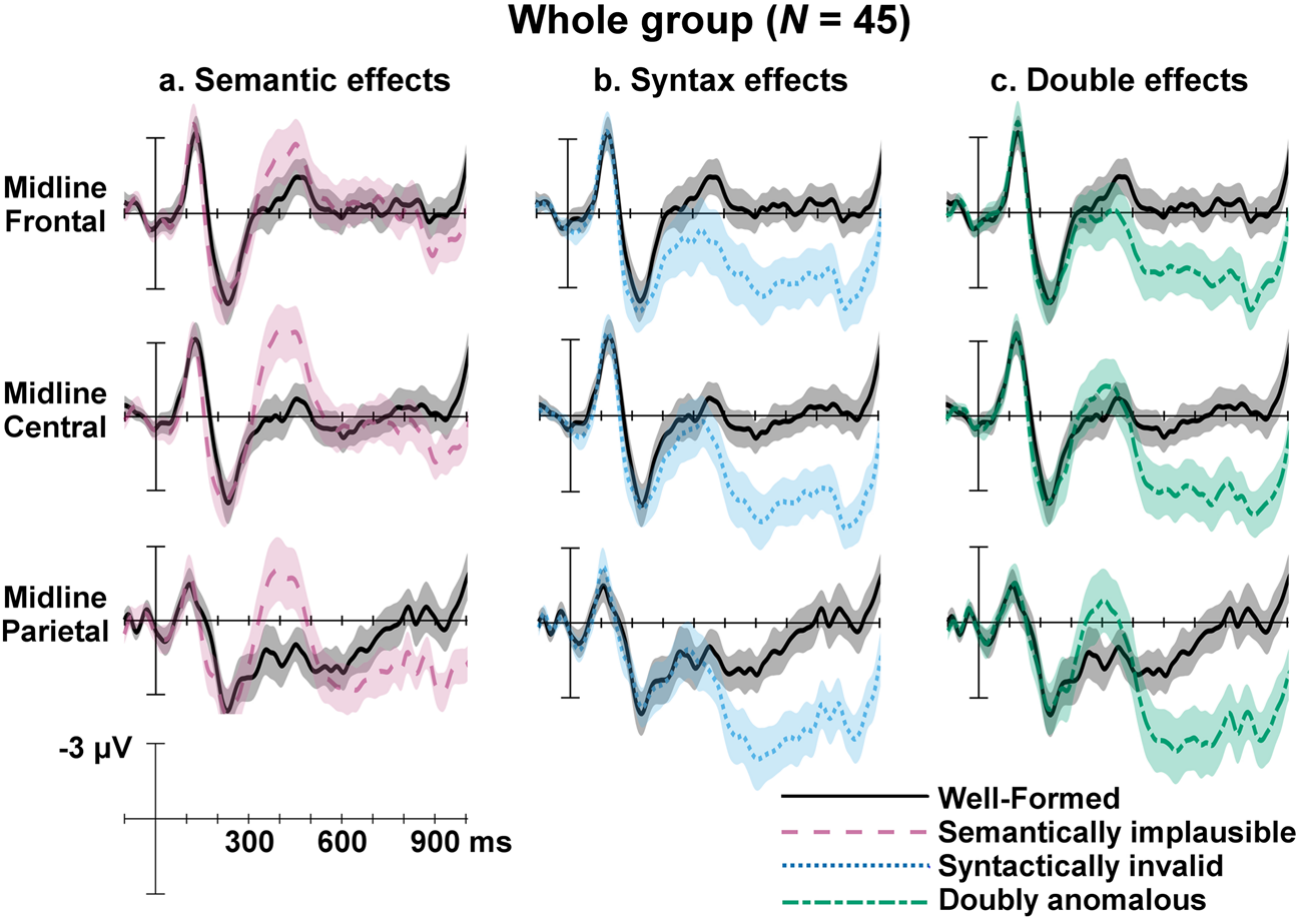
Grand mean ERP waveforms for all participants (*N* = 45). Well-formed code (black, solid) is compared to (**a**) semantically implausible code (pink, dashed), (**b**) syntactically invalid code (blue, dotted), and (**c**) doubly anomalous code (green, dash-dotted). The shaded region around the ERP waveforms indicate the 95% confidence interval. Onset of the target item of code in a given trial is indicated by the vertical bar. Calibration bar shows 3 μV of activity. Negative voltage is plotted up.

Group-level ERP responses for semantic plausibility (Fig. 3A), syntactic validity (Fig. 3B), and double anomalies (Fig. 3C) are plotted separately in Fig. 3.

#### N400 (300–500 ms) time window

The overall model predicting ERP responses within the N400 time window has a total explanatory power of 59.93%, in which the fixed effects explain 13.14% of the variance. The model’s intercept is at − 0.02 (*SE* = 0.39, 95% CI [− 0.78, 0.74], *t*(43) = − 0.05, *p* = 0.964). Within this model, the effect of semantic plausibility was significant in predicting the N400 response (*b* = − 1.71, *SE* = 0.20, 95% CI [− 2.11, − 1.32], *t*(488) = − 8.58, *p* < 0.001). Code containing semantic implausibility resulted in larger negative deflections than did code without semantic implausibility. There was also a significant effect of syntactic validity, with syntactic anomalies resulting in larger positive deflections than well-formed code (*b* = 1.40, *SE* = 0.20, 95% CI [1.01, 1.70], *t*(488) = 7.01, *p* < 0.001). As is apparent in Fig. 3B, this effect was likely driven by the later P600 effect described in the next section. Specifically, positive deflections to syntactically invalid code in the P600 time window were large enough to be detectable within the earlier N400 time window. There was no significant interaction between semantic plausibility and syntactic validity. However, there was a significant effect of electrode, with the effect being driven by more negative deflections at the midline central electrode than at the midline parietal electrode (*b* = − 1.18, *SE* = 0.24, 95% CI [− 1.66, − 0.70], *t*(488) = − 4.81, *p* < 0.001). Full model results for all fixed effects and random components are reported in Supplementary Tables S4–S6.

#### P600 (500–800 ms) time window

The overall model predicting ERP responses within the P600 time window has a total explanatory power of 56.76%, in which the fixed effects explain 31.87% of the variance. The model’s intercept is at 2.07 (*SE* = 0.28, 95% CI [1.52, 2.62], *t*(43) = 7.35, *p* < 0.001). Within this model, the effect of syntactic validity was significant in predicting the P600 response (*b* = 2.97, *SE* = 0.20, 95% CI [2.58, 3.36], *t*(488) = 14.84, *p* < 0.001). Code containing syntactic anomalies resulted in larger positive deflections than code without syntactic anomalies. There was no significant effect of semantic plausibility or significant interaction between semantic plausibility and syntactic validity in the P600 window. However, there was a significant effect of electrode, with all three electrode sites differing in amplitudes from each other. There were more positive deflections recorded at the midline frontal electrode than at the midline central electrode (*b* = 0.62, *SE* = 0.25, 95% CI [0.14, 1.10], *t*(488) = 2.54, *p* = 0.011), which in turn recorded more positive deflections than the midline parietal electrode (*b* = 1.50, *SE* = 0.25, 95% CI [1.01, 1.98], *t*(488) = 1.71, *p* < 0.001). Full model results for all fixed effects and random components are reported in Supplementary Tables S7–S9.

### Effects of expertise on ERP responses

**Figure 4:**
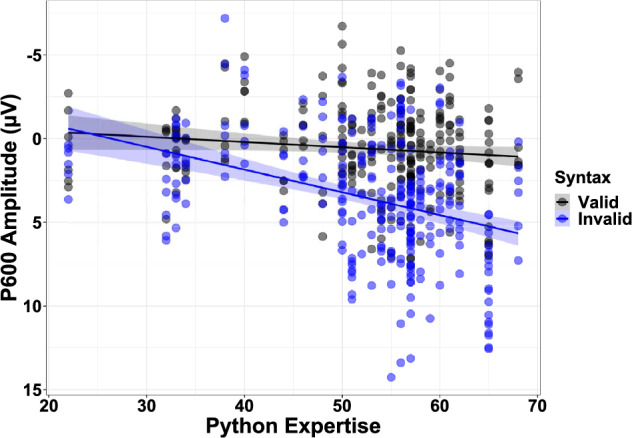
Amplitude of P600 responses (μV) to code with (blue) and without (black) syntactic anomalies, collapsed across semantic plausibility and electrodes, as a function of Python expertise (% on a Python knowledge test). Negative voltage is plotted up to mirror the traditional ERP plotting method. The shaded region around the trend lines indicate the 95% confidence interval.

Python expertise, along with its interaction with semantic plausibility and syntactic validity, were also included as fixed effects in the models predicting N400 and P600 responses. Within the P600 window, Python expertise significantly predicted ERP responses (*b* = 0.83, *SE* = 0.28, 95% CI [0.28, 1.38], *t*(43) = 2.94, *p* = 0.005). There was also a significant interaction between Python expertise and syntactic validity (*b* = 1.04, *SE* = 0.20, 95% CI [0.64, 1.43], *t*(488) = 5.18, *p* < 0.001), but not semantic plausibility. With increasing expertise, programmers exhibited a stronger P600 effect to code containing syntactic anomalies than to code without syntactic anomalies (Fig. 4). There was also a significant interaction between Python expertise and syntactic validity within the N400 window (*b* = 0.45, *SE* = 0.20, 95% CI [0.06, 0.84], *t*(488) = 2.24, *p* = 0.025) that was likely driven by the same interaction within the P600 window. With increasing expertise, programmers exhibited more positive deflections to syntactically invalid code in the P600 time window, causing these effects to be more detectable within the earlier N400 time window. Full details of the effect of Python expertise on ERP responses are reported in Supplementary Tables 4–5 (N400 window) and 7–8 (P600 window).

**Figure 5:**
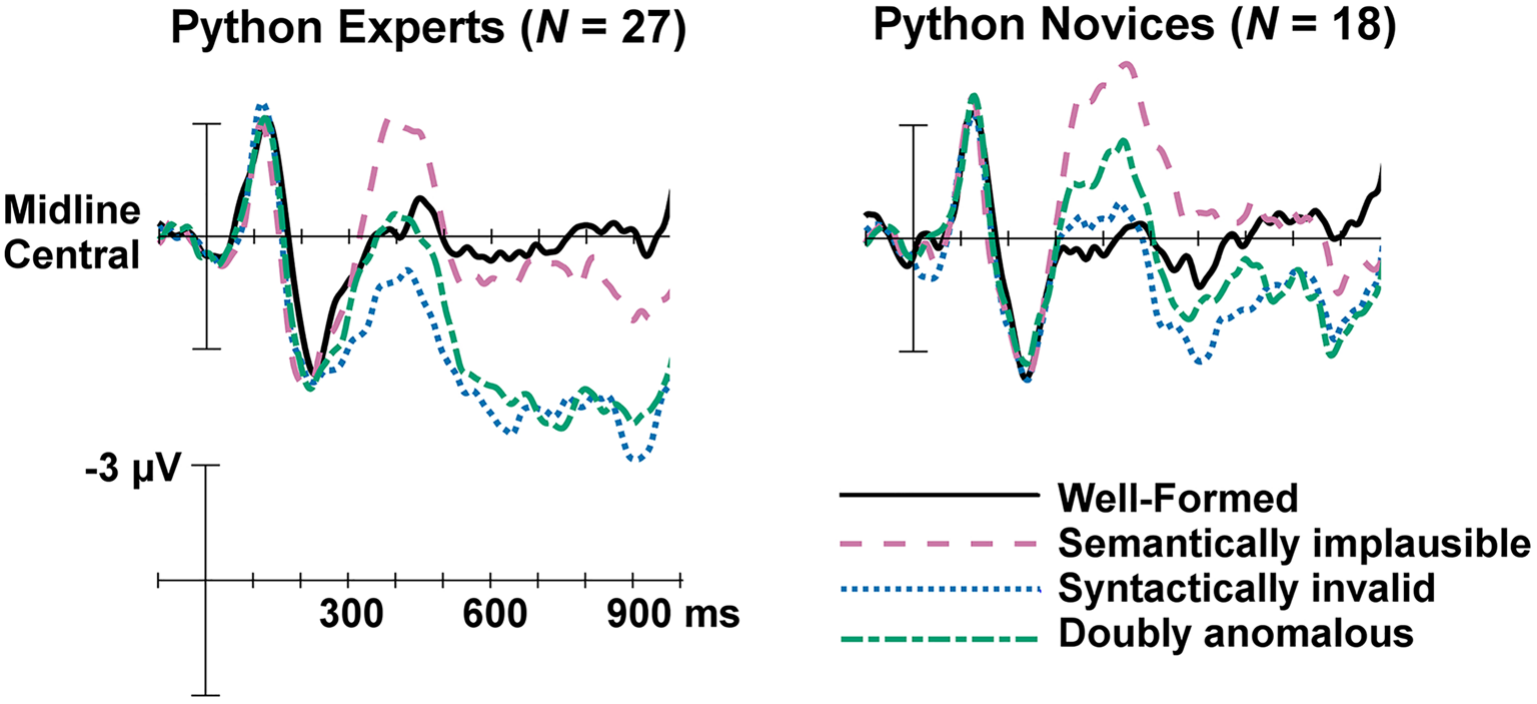
Summary of the ERP waveforms for Python experts (*N* = 27) versus Python novices (*N* = 18) for all four conditions at the midline central electrode. Onset of the target item of code in a given trial is indicated by the vertical bar. Calibration bar shows 3 μV of activity. Negative voltage is plotted up.

For visualization purposes, we also plotted the mean ERP responses of Python experts (*N* = 27) and Python novices (*N* = 18) at the midline central electrode in Fig. 5. Python knowledge test accuracies (see "Methods") were used to determine the two groups of Python expertise. Participants who scored 75% or higher on the Python knowledge test were considered experts, whereas participants who scored less than 75% were considered novices. 75% corresponds to a 2.0 on a standard 4.0 GPA scale, which is the lowest passing grade allowed for some courses with more stringent grading policies at the University of Washington, where this experiment was conducted.

## Discussion

To the best of our knowledge, this is the first empirical data supporting the cognitive comparisons between reading and code comprehension first outlined by Fedorenko et al.^6^. Specifically, our data suggest that when skilled programmers read lines of code, they use information about both statement-level form and token-level meaning to incrementally update their mental representation of what the code is trying to accomplish, much like readers of a natural language use information about grammatical structure and word-level semantics to understand what a sentence means. In our experiment, this was reflected by the distinct N400 and P600 effects exhibited by programmers to semantic and syntactic manipulations of code, respectively.

Furthermore, results from this study suggest that as programmers gain expertise in a particular programming language, their brain responses increasingly reflect sensitivity to rule-based knowledge in their online comprehension processes. This progression, termed “grammaticalization” in natural language learning, is also observed with increasing proficiency in second natural language acquisition^15^. Although our analysis of expertise was cross-sectional, it is notable that this pattern of increased P600 effects with higher expertise has been demonstrated in both cross-sectional^16^ and longitudinal^15^ studies of learners of a second natural language. As such, we would expect to see a similar trend in which stronger P600 effects would emerge over time with increasing exposure to programming languages. We see this as an interesting area for future research.

It is worth noting that programming expertise may not have influenced neural sensitivity to semantic manipulations, but the manner in which we manipulated semantic plausibility reflected pre-existing semantic relations (e.g., categorical relations among variable names) rather than code-specific relations (e.g., substituting functions that are related to, but not the intended, operation in a line of code). Though our semantic manipulations are far from a complete exploration, similar categorical manipulations have been used to understand the cognitive underpinnings of mathematical comprehension^14^ both at the group and individual levels. Additionally, these findings suggest that programmers of varying expertise levels show neural sensitivity to pre-existing semantic relations in lines of code, even when they have little to no relevance for what the code does (e.g., in variable names). This additional neural activation has been proposed to reflect increased difficulty in either retrieving the meaning of the target item^12,19,20^ or integrating that meaning into the overall representation of the structure at hand^21^. The behavioral trend we observed also suggests that less skilled programmers, whose rule-based processes have not fully come online yet, are marginally more likely to judge a well-formed line of code as unacceptable when the semantic relations among items are implausible. Taken together, these data support the notion that using pre-existing meaning associations can facilitate code comprehension for some individuals. This is consistent with previous work demonstrating that meaningful and efficient identifiers promote faster and more accurate code comprehension^22–24^.

When interpreting these results, we note that the observation of language-like responses to violations in lines of code does not, in and of itself, provide evidence that code comprehension relies on the *same* neural substrates as language comprehension. Such an inference would require a tool with better spatial resolution, such as fMRI, and the results from such studies have been mixed^1,2,8^. Instead, these results contribute to the growing body of work indicating that the N400 and P600 components are not *specific* to natural language processing^12–14,25,26^. Findings from research employing diverse stimuli including mathematical word problems^14^, natural language^12,13^, music^25,26^, and now Python code converge to demonstrate common neurocomputations that lie at the heart of integrating sequential information incrementally into a larger meaning structure. In light of these similarities, we propose that the current debate about whether code is more “language-like” or “mathematics-like” might be better framed by questioning the types of information programmers use to understand what a line of code does, and how this evolves as they become more proficient.

In summary, we present the first study showcasing that programmers exhibit neural sensitivity to information about form and meaning as they engage in real-time incremental building of mental representations during code comprehension. When doing so, expert programmers are more sensitive to the structural relations among items, characterized by prominent brain responses to syntactic violations within 600 ms of seeing an item. In contrast, programmers of varying skill levels exhibit similar neural sensitivity to the pre-existing semantic relations among items in the code, with more novice programmers showing marginally greater reliance on such information when making offline behavioral “acceptability” judgments. Taken together, these results suggest that the processes that support code comprehension resemble those of other learned symbolic, rule-based systems such as reading, algebra, and classroom-based L2 learning.

## Methods

### Participants

Sixty-two right-handed individuals aged 18–33 years with normal or corrected-to normal vision and no history of significant head injury or epilepsy were recruited for participation in this study. All participants had a minimum of the equivalent of one academic quarter’s worth of Python instruction, either through a live course taught by an instructor or self-taught via an online course. One individual did not return for the ERP session and was removed from all analyses. Of the remaining 61 individuals, 16 exceeded the maximum 25% rejection rate threshold for their averaged ERPs and were removed from all analyses (see Data analysis). The final sample consisted of 45 English-speaking participants (23 female, 21 male, 1 other) from various natural language backgrounds. One participant wore hearing aids during EEG recording. For additional participant demographics, see Supplementary Table S10. All experimental procedures were approved by the University of Washington Institutional Review Board and performed in accordance with relevant guidelines and regulations. Participants gave informed consent prior to the start of the experiment and were monetarily compensated for their time.

### Materials

#### Python knowledge test

Participants completed a 72-question multiple-choice test as a quantitative measurement of their Python proficiency^3^. Half of the questions measured semantic knowledge, such as the purpose of functions and operators (e.g., “What does the print() function do?”). The other half measured knowledge about syntax, or structural rules of Python (e.g., “Why won’t the following code compile?”). This measure was developed as part of another study that assessed learners’ Python knowledge following weekly lessons in the Python 2 course on Codecademy^7^. As such, the questions were created according to the material covered, including, but not limited to strings, conditionals, functions, lists, dictionaries, and loops. Python proficiency was quantified as a percentage accuracy score by dividing the total number of questions correct by 72 total possible questions. For Python knowledge test results, see Supplementary Table S10.

#### ERP stimuli

Lines of Python code were created by crossing semantic plausibility and syntactic validity in a two by two design. A unique global variable preceded every line of code, which provided thematic context for an individual trial. Global variables could be strings, integers, floats, lists, or dictionaries. Each line of code contained a single item that was either semantically plausible or semantically implausible given the global variable, as well as either syntactically valid or syntactically invalid according to the syntax rules of Python 3.0. This resulted in four different code conditions: (1) well-formed, (2) semantically implausible, (3) syntactically invalid, and (4) doubly anomalous (semantically implausible and syntactically invalid). All lines of code spanned five to nine item lengths with the violation position occurring at two, three, or four.

To provide variability in stimuli, two code structures were used in this experiment: for loops and list comprehensions (Table 1). Violations were created by manipulating one of two code types: variables or keywords. Variables are placeholder words (i.e., iterators) that parse through each item of an iterable, such as a list or a dictionary. Although variables can be named using any combination of letters and numbers, it is common practice to give them an identifier that is thematically consistent with the object being iterated through. For example, if referencing the list *pets* = *[“dog”, “cat”, “hamster”]*, a variable named *animal* (e.g., *for every animal in the list pets…*) rather than *fruit* (e.g., *for every fruit in the list pets…*) would be more semantically appropriate for iterating through each list item. However, variables cannot be attached to operators or symbols, such as quotations that are used to signify a string. Therefore, a variable written as *animal* rather than *“animal”* (e.g., *for every “animal” in the list pets…*) would be the syntactically appropriate form. As such, variables can be manipulated to be semantically implausible, syntactically invalid, or doubly anomalous (e.g., *for every “fruit” in the list pets…*). On the contrary, keywords are reserved words that have specific roles, and they cannot be used as variable names, function names, or other identifiers. The present study manipulated two keywords, *if* and *in*, by replacing them with other English words that were either approximate synonyms or semantically dissimilar. For example, the keyword *in* could be replaced with *within* (an approximate synonym) or *under* (semantically dissimilar). However, because keywords are built-in, they cannot be manipulated to be semantically implausible while remaining syntactically valid according to Python syntax rules. As such, keywords can only be manipulated to be syntactically invalid (e.g., *for every animal within the list pets…*) or doubly anomalous (e.g., *for every animal under the list pets…*). In order to accommodate for this imbalance, additional lines of code that manipulated the variable were written to allow for a balanced, fully crossed two by two design.

**Table 1:** Stimulus examples.

Condition	Example For Loop	Example List Comprehension
*Global variable*	pets = [“dog”, “cat”, “hamster”]	felines = [“lion”, “tiger”, “leopard”]
Well-formed	for animal in pets: print(animal)	[“purr” for cat in felines if cat = = “tiger”]
Semantically implausible	for fruit in pets: print(fruit)	[“purr” for wheel in felines if cat = = “tiger”]
Syntactically invalid	for “animal” in pets: print(animal)	[“purr” for “cat” in felines if cat = = “tiger”]
Doubly anomalous	for “fruit” in pets: print(fruit)	[“purr” for “wheel” in felines if cat = = “tiger”]

The critical piece of code for ERP averaging is underlined. The second row indicates the global variable that is shown prior to that given example stimulus.

For the lines of code in which it was possible, the four versions corresponding to each condition were distributed across four experimental lists, such that each list only had one version of each line of code. Participants saw 160 lines of code, with 40 lines from each condition. In the well-formed, syntactically invalid, and doubly anomalous conditions, the 40 lines of code were split between 20 manipulations of variables and 20 manipulations of keywords. In the semantically implausible condition, all 40 lines of code were manipulations of variables. Each list was divided into 4 blocks of 40 sentences each, and each block contained 10 lines of code from each condition. The 20 additional lines of code written to round out the semantically implausible condition were included in each list. Lines of code were pseudo-randomized within each list, and list assignment was pseudo-randomized across participants. Complete stimulus lists can be found on OpenNeuro in the Stimulus folder (see Data availability).

### Procedure

#### Main experiment

Participants took part in two sessions, each lasting no more than two hours. With the exception of one individual, all participants completed the experiment in the same session order. Session 1 was administered over videoconference, during which participants completed all questionnaires and tasks related to demographics, natural language background, and programming experience. During Session 2 on a separate day, participants judged individual lines of Python code for acceptability while electroencephalogram recordings were obtained. After being seated in a desk chair in front of a CRT monitor, participants were instructed to relax and minimize movements and eye blinks while silently reading the stimuli in their minds. Each trial consisted of the following events: Participants were given 15 s to read the global variable to be referenced in the upcoming line of code, or they could proceed earlier by clicking a mouse button. Following a 1000 ms fixation cross and 200 ISI, the line of code appeared incrementally in the center of the screen one item at a time, as defined by blank space, at a presentation rate of 700 ms and 200 ms ISI. These slower presentation rates are standard for ERP studies of a second language, for both native speakers and learners^16,17,26–28^ (see Supplementary Methods for details of the behavioral pilot study that was conducted to determine optimal presentation rate). After the line of code was finished displaying, a “Yes/No” screen followed, during which participants had 30 s to give their acceptability judgment by pressing one of two mouse buttons. “Yes” corresponded to lines of code that were acceptable, and “No” corresponded to lines of code that were unacceptable. Participants were asked to use their own criteria for what they considered to be “acceptable” and to keep this criteria consistent throughout the session. The order of the “Yes/No” response buttons (left/right) was pseudo-randomized across participants. Once a response was given, a “READY?” prompt appeared, and participants would click either mouse button to begin the next trial.

### Data analysis

#### Behavioral code acceptability judgment task

Behavioral performance on the ERP task was assessed via acceptability judgment rates, which were calculated as the percentage of trials that participants considered to be “acceptable,” i.e., responded with “Yes” during the end-of-code judgment task. We ran a linear mixed model fit by restricted maximum likelihood (REML) using the GAMLj module in Jamovi^29–31^. Acceptability rates (“Acceptability”) were predicted from fixed effects of semantic plausibility (“Semantics”), syntactic validity (“Syntax”), and Python expertise (“Expertise”). Two levels of semantic plausibility (semantically plausible, semantically implausible) and two levels of syntactic validity (syntactically valid, syntactically invalid) accounted for the four code conditions. Python expertise was entered as a participant’s z-transformed Python knowledge test score. Interactions between semantic plausibility and syntactic validity, semantic plausibility and Python expertise, and syntactic validity and Python expertise were also included in the model as fixed effects. Participants (“Subject”) were included as a random effect in which the intercept was permitted to vary. Altogether, the model specification was as follows: Acceptability ~ 1 + Semantics + Syntax + Expertise + Semantics:Syntax + Semantics:Expertise + Syntax:Expertise + (1 | Subject). The Satterthwaite method was used to estimate degrees of freedom and generate *p*-values.

#### EEG acquisition

Continuous EEG was recorded from 32 scalp electrodes placed in International 10–20 system locations attached to a Biosemi elastic cap^32^. Eye movements and blinks were monitored by two electrodes placed beneath the left eye and to the right of the right eye. Electrodes were referenced to an electrode placed over the left mastoid during recording, then subsequently re-referenced to the average of two electrodes placed over the left mastoid and right mastoid during pre-processing. EEG signals were amplified with a bandpass filter of 0.01 to 30 Hz by a Biosemi bioamplifier system. Impedances at scalp electrodes were held below 50 Hz. Continuous analog-to-digital conversion of the EEG and stimulus trigger codes was performed at a sampling frequency of 200 Hz.

#### EEG cleaning

For each participant, epochs of EEG signal were segmented around the critical item of code from − 100 ms to 1205 ms. Epochs were removed from ERP averaging if they contained changes within a sliding 200 ms window that were greater than 100 µV in the midline central electrode. Trials characterized by excessive eye movement, muscle artifact, and alpha were further removed prior to averaging; these were epochs that showed voltage steps more extreme than − 65 µV or 65 µV within any electrode. Participants who had a > 25% artifact rejection rate were removed from analysis altogether, resulting in the final sample of 45 individuals. The average rejection rate for participants who were included in the final analyses was 6.8% (by condition: well-formed: 7.5%, semantically implausible: 6.4%, syntactically invalid: 6.9%, doubly anomalous: 6.4%). Due to a malfunction in the recording equipment, two participants in the final analyses had fewer than 160 trials (159 and 158 trials); both individuals averaged rejection rates of 3.8%.

#### EEG analysis

ERPs time-locked to the onset of the critical item of code in each line of code were averaged offline at each electrode site in each condition. The epochs that went into ERP averaging were base-line corrected from − 100 ms to 0 ms. As is standard, the following time windows were chosen for analysis: 300–500 ms (N400) and 500–800 ms (P600), with separate analyses conducted for each window. All trials (both acceptable and not acceptable judgment responses) were included in the final analysis. This decision was made for two reasons: First, previous research has shown that neural sensitivity to anomalies sometimes precedes behavioral sensitivity in L2 learners^33^, and second, the definition of “acceptable” was intentionally made ambiguous so that learners could decide how to treat semantically anomalous trials. This made deciding whether trials were correct or incorrect difficult in some conditions.

We ran linear mixed models fit by restricted maximum likelihood (REML) using the GAMLj module in Jamovi^29–31^. ERP responses (“Amplitude”) were predicted from fixed effects of semantic plausibility (“Semantics”), syntactic validity (“Syntax”), Python expertise (“Expertise”), and electrode (“Electrode”) for each time window. As in the model predicting acceptability judgments, two levels of semantic plausibility (semantically plausible, semantically implausible) and two levels of syntactic validity (syntactically valid, syntactically invalid) accounted for the four code conditions. Python expertise was entered as a participant’s z-transformed Python knowledge test score. We had no hypotheses that were centered on scalp topography; therefore, we included data from the midline frontal, central, and parietal electrodes as three levels of electrode. Interactions between semantic plausibility and syntactic validity, semantic plausibility and Python expertise, and syntactic validity and Python expertise were also included in the model as fixed effects. Participants (“Subject”) were included as a random effect in which the intercept was permitted to vary. Altogether, the model specification for both time windows was as follows: Amplitude ~ 1 + Semantics + Syntax + Expertise + Electrode + Semantics:Syntax + Semantics:Expertise + Syntax:Expertise + (1 | Subject). The Satterthwaite method was used to estimate degrees of freedom and generate *p*-values.

We also conducted another set of linear mixed models that added the fixed effect of English proficiency (“English”), as well as its interactions with semantic plausibility and syntactic validity, to see if we could better predict code acceptability judgments and ERP responses. English proficiency was determined via a participant’s score on the Nelson-Denny Reading Comprehension test^34^ and was entered into the models after z-transformation. The full results of the models that added English proficiency are reported in Supplementary Tables S11–S13 (code acceptability judgments), S14–S16 (N400 window), and S17–S19 (P600 window). Comparison of BIC values showed that these models did not improve upon the models that did not include English proficiency (Supplementary Table S20). As such, we focused our discussion on the implications of Python expertise.

### Supplementary Information

Supplementary Information.

## Data Availability

All data is available in the manuscript, supplementary materials, or on OpenNeuro: https://openneuro.org/datasets/ds004771.
