# Supplementary material for: Computer programmers show distinct, expertise-dependent brain responses to violations in form and meaning when reading code
Source: Sci Rep. 2024 Mar 5;14:5404. doi: 10.1038/s41598-024-56090-6 (PMC10914777; doi:10.1038/s41598-024-56090-6)
Supplement: Supplementary file 1 — Supplementary Information. [file 41598_2024_56090_MOESM1_ESM.pdf]

Supplementary Information for:

Computer programmers show distinct, expertise-dependent brain responses to violations in form and meaning when reading code

Chu-Hsuan Kuo[1]* and Chantel S. Prat[1,2]

[1]Department of Psychology, University of Washington, Seattle, Washington, USA.
[2]Institute for Learning and Brain Sciences, University of Washington, Seattle, Washington, USA.
*email: kuoc6@uw.edu

Supplementary Methods

Pilot studies. Due to the novelty of presenting code in incremental format, behavioral pilot studies were run to determine the presentation rate that would maximize performance on the task and minimize working memory demands. In the first pilot study, a list of 160 lines of code with four blocks were constructed as described in the ERP stimuli section of the manuscript. This list was coded as a behavioral experiment using the PsyToolkit platform[1,2] to resemble the incremental format used for the main ERP experiment. Two blocks used a presentation rate of 600 ms and 200 ms ISI, and two blocks used a presentation rate of 700 ms and 200 ms ISI. Three participants with varying levels of Python expertise completed the acceptability judgment task at a computer. After comparing the acceptability judgments of the 600 ms blocks to the 700 ms blocks across the participants, it was determined that the responses were similar between the two presentation rates, but the accuracies for the well-formed conditions were higher for the 700 ms blocks. Therefore, a presentation rate of 700 ms and 200 ms ISI was chosen for a second ERP pilot study, which was conducted according to the procedures described for the main experiment. Four participants with varying levels of Python expertise completed the ERP pilot study in full. Visual inspection of their averaged ERP waveforms revealed prominent deflections in the N400 and P600 time windows that suggested sensitivity to the semantic and syntactic manipulations. As such, a presentation rate of 700 ms and 200 ms ISI was selected for the main experiment.

Supplementary Table S1.
Tests of fixed effects for the linear mixed model predicting code acceptability judgments.

**Source**	***F***	**Num *df***	**Den *df***	***p***
*Semantics	18.84	1.00	130.00	<.001
*Syntax	356.13	1.00	130.00	<.001
Expertise	0.24	1.00	43.00	.628
Semantics:Syntax	0.12	1.00	130.00	.728
Semantics:Expertise	3.88	1.00	130.00	.051
*Syntax:Expertise	71.76	1.00	130.00	<.001

Note. Semantics = semantic plausibility; Syntax = syntactic validity; Expertise = Python expertise. Due to the categorical nature of all fixed effects except for Expertise, parameter estimates are unstandardized; however, Expertise was *z*-transformed prior to being entered into the model. The Satterthwaite method was used to estimate degrees of freedom and generate *p*-values. *significant at an alpha of .05.

Supplementary Table S2.
Estimates of fixed effects for the linear mixed model predicting code acceptability judgments.

			95% CI				
**Parameter**	**Estimate**	***SE***	**Lower**	**Upper**	***df***	***t***	***p***
*Intercept	45.59	1.96	41.75	49.43	43.00	23.29	<.001
*Semantics	11.66	2.69	6.39	16.92	130.00	4.34	<.001
*Syntax	50.67	2.69	45.41	55.94	130.00	18.87	<.001
Expertise	-0.96	1.96	-4.81	2.89	43.00	-0.49	.628
Semantics:Syntax	1.87	5.37	-8.65	12.40	130.00	0.35	.728
Semantics:Expertise	-5.30	2.69	-10.58	-0.03	130.00	-1.97	.051
*Syntax:Expertise	22.81	2.69	17.53	28.09	130.00	8.47	<.001

Note. Semantics = semantic plausibility; Syntax = syntactic validity; Expertise = Python expertise. Due to the categorical nature of all fixed effects except for Expertise, parameter estimates are unstandardized; however, Expertise was z-transformed prior to being entered into the model. The Satterthwaite method was used to estimate degrees of freedom and generate *p*-values. *significant at an alpha of .05

Supplementary Table S3.
Random components for the linear mixed model predicting code acceptability judgments.

**Effect**	**Name**	***SD***	**Variance**	**ICC**
Subject	(Intercept)	9.56	91.33	0.22
Residual		18.01	324.47	

Supplementary Table S4.
Tests of fixed effects for the linear mixed model predicting ERP responses in the N400 window.

**Source**	***F***	**Num *df***	**Den *df***	***p***
*Semantics	73.63	1.00	488.00	<.001
*Syntax	49.14	1.00	488.00	<.001
Expertise	1.43	1.00	43.00	.239
*Electrode	12.46	2.00	488.00	<.001
Semantics:Syntax	2.53	1.00	488.00	.112
Semantics:Expertise	0.02	1.00	488.00	.887
*Syntax:Expertise	5.04	1.00	488.00	.025

Note. Semantics = semantic plausibility; Syntax = syntactic validity; Expertise = Python expertise. Due to the categorical nature of all fixed effects except for Expertise, parameter estimates are unstandardized; however, Expertise was z-transformed prior to being entered into the model. The Satterthwaite method was used to estimate degrees of freedom and generate *p*-values. *significant at an alpha of .05.

Supplementary Table S5.
Estimates of fixed effects for the linear mixed model predicting ERP responses in the N400 window.

			95% CI				
**Parameter**	**Estimate**	***SE***	**Lower**	**Upper**	***df***	***t***	***p***
Intercept	-0.02	0.39	-0.78	0.74	43.00	-0.05	.964
*Semantics	-1.71	0.20	-2.11	-1.32	488.00	-8.58	<.001
*Syntax	1.40	0.20	1.01	1.79	488.00	7.01	<.001
Expertise	0.46	0.39	-0.30	1.22	43.00	1.19	.239
Electrode (Fz - Cz)	0.31	0.24	-0.17	0.79	488.00	1.26	.208
*Electrode (Cz - Pz)	-1.18	0.24	-1.66	-0.70	488.00	-4.81	<.001
Semantics:Syntax	0.64	0.40	-0.15	1.42	488.00	1.59	.112
Semantics:Expertise	0.03	0.20	-0.36	0.42	488.00	0.14	.887
*Syntax:Expertise	0.45	0.20	0.06	0.84	488.00	2.24	.025

Note. Semantics = semantic plausibility; Syntax = syntactic validity; Expertise = Python expertise; Fz = midline frontal; Cz = midline central; Pz = midline parietal. Due to the categorical nature of all fixed effects except for Expertise, parameter estimates are unstandardized; however, Expertise was z-transformed prior to being entered into the model. The Satterthwaite method was used to estimate degrees of freedom and generate *p*-values.
*significant at an alpha of .05

Supplementary Table S6.
Random components for the linear mixed model predicting ERP responses in the N400 window.

**Effect**	**Name**	***SD***	**Variance**	**ICC**
Subject	(Intercept)	2.51	6.29	0.54
Residual		2.32	5.39	

Supplementary Table S7.

Tests of fixed effects for the linear mixed model predicting ERP responses in the P600 window.

**Source**	***F***	**Num *df***	**Den *df***	***p***
Semantics	0.09	1.00	488.00	.761
*Syntax	220.11	1.00	488.00	<.001
*Expertise	8.64	1.00	43.00	.005
*Electrode	39.45	2.00	488.00	<.001
Semantics:Syntax	2.93	1.00	488.00	.088
Semantics:Expertise	0.01	1.00	488.00	.907
*Syntax:Expertise	26.79	1.00	488.00	<.001

Note. Semantics = semantic plausibility; Syntax = syntactic validity; Expertise = Python expertise. Due to the categorical nature of all fixed effects except for Expertise, parameter estimates are unstandardized; however, Expertise was z-transformed prior to being entered into the model. The Satterthwaite method was used to estimate degrees of freedom and generate *p*-values. *significant at an alpha of .05.

Supplementary Table S8.
Estimates of fixed effects for the linear mixed model predicting ERP responses in the P600 window.

			**95% CI**				
**Parameter**	**Estimate**	***SE***	**Lower**	**Upper**	***df***	***t***	***p***
*Intercept	2.07	0.28	1.52	2.62	43.00	7.35	<.001
Semantics	-0.06	0.20	-0.45	0.33	488.00	-0.30	.761
*Syntax	2.97	0.20	2.58	3.36	488.00	14.84	<.001
*Expertise	0.83	0.28	0.28	1.38	43.00	2.94	.005
*Electrode (Cz - Fz)	0.62	0.25	0.14	1.10	488.00	2.54	.011
*Electrode (Pz - Cz)	1.50	0.25	1.01	1.98	488.00	6.10	<.001
Semantics:Syntax	-0.69	0.40	-1.47	0.10	488.00	-1.71	.088
Semantics:Expertise	-0.02	0.20	-0.42	0.37	488.00	-0.12	.907
*Syntax:Expertise	1.04	0.20	0.64	1.43	488.00	5.18	<.001

Note. Semantics = semantic plausibility; Syntax = syntactic validity; Expertise = Python expertise; Fz = midline frontal; Cz = midline central; Pz = midline parietal. Due to the categorical nature of all fixed effects except for Expertise, parameter estimates are unstandardized; however, Expertise was z-transformed prior to being entered into the model. The Satterthwaite method was used to estimate degrees of freedom and generate *p*-values.
*significant at an alpha of .05

Supplementary Table S9.
Random components for the linear mixed model predicting ERP responses in the P600 window.

**Effect**	**Name**	***SD***	**Variance**	**ICC**
Subject	(Intercept)	1.76	3.11	0.37
Residual		2.33	5.41	

Supplementary Table S10.
Descriptive statistics for participant demographic information.

**Demographic Measure**	**Mean**	***SD***	**Min**	**Max**
Age of first exposure to English	2.07	2.7	0	9
Age achieving general fluency	7.02	5.8	0	24
Overall self-rated English proficiency (0-10)	9.3	1.09	6.33	10
Nelson-Denny reading comprehension test (%)	81.81	17.15	26.32	100
Python knowledge test (%)	73.09	13.91	30.56	94.44

Note. Demographics were calculated for the final sample of 45 participants.

Supplementary Table S11.
Tests of fixed effects for the linear mixed model predicting code acceptability judgments, with the inclusion of English proficiency as a predictor.

**Source**	***F***	**Num *df***	**Den *df***	***p***
*Semantics	18.57	1.00	128.00	<.001
*Syntax	250.98	1.00	128.00	<.001
Expertise	0.006	1.00	42.00	.940
English	0.60	1.00	42.00	.442
Semantics:Syntax	0.12	1.00	128.00	.730
Semantics:Expertise	3.28	1.00	128.00	.072
*Syntax:Expertise	57.93	1.00	128.00	<.001
Semantics:English	0.03	1.00	128.00	.870
Syntax:English	0.09	1.00	128.00	.760

Note. Semantics = semantic plausibility; Syntax = syntactic validity; Expertise = Python expertise; English = English proficiency. Due to the categorical nature of Semantics and Syntax, parameter estimates are unstandardized; however, Expertise and English were z-transformed prior to being entered into the model. The Satterthwaite method was used to estimate degrees of freedom and generate *p*-values. *significant at an alpha of .05.

Supplementary Table S12.
Estimates of fixed effects for the linear mixed model predicting code acceptability judgments, with the inclusion of English proficiency as a predictor.

			95% CI				
**Parameter**	**Estimate**	***SE***	**Lower**	**Upper**	***df***	***t***	***p***
*Intercept	45.59	1.97	41.73	49.44	42.00	23.18	<.001
*Semantics	11.66	2.70	6.36	16.96	128.00	4.31	<.001
*Syntax	50.67	2.70	45.37	55.98	128.00	18.73	<.001
Expertise	-0.17	2.22	-4.52	4.18	42.00	-0.08	.940
English	-1.72	2.22	-6.08	2.63	42.00	-0.78	.442
Semantics:Syntax	1.87	5.41	-8.73	12.48	128.00	0.35	.730
Semantics:Expertise	-5.53	3.05	-11.52	-.45	128.00	-1.81	.072
*Syntax:Expertise	23.24	3.05	17.25	29.22	128.00	7.61	<.001
Semantics:English	0.50	3.05	-5.48	6.49	128.00	0.16	.870
Syntax:English	-0.93	3.05	-6.92	5.05	128.00	-0.31	.760

Note. Semantics = semantic plausibility; Syntax = syntactic validity; Expertise = Python expertise; English = English proficiency. Due to the categorical nature of Semantics and Syntax, parameter estimates are unstandardized; however, Expertise and English were z-transformed prior to being entered into the model. The Satterthwaite method was used to estimate degrees of freedom and generate *p*-values. *significant at an alpha of .05.

Supplementary Table S13.
Random components for the linear mixed model predicting code acceptability judgments, with the inclusion of English proficiency as a predictor.

**Effect**	**Name**	***SD***	**Variance**	**ICC**
Subject	(Intercept)	9.58	91.73	0.22
Residual		18.14	329.23	

Supplementary Table S14.
Tests of fixed effects for the linear mixed model predicting ERP responses in the N400 window, with the inclusion of English proficiency as a predictor.

**Source**	***F***	**Num *df***	**Den *df***	***p***
*Semantics	74.37	1.00	486.00	<.001
*Syntax	49.64	1.00	486.00	<.001
Expertise	1.00	1.00	42.00	.323
English	0.01	1.00	42.00	.917
*Electrode	12.59	2.00	486.00	<.001
Semantics:Syntax	2.55	1.00	486.00	.111
Semantics:Expertise	0.81	1.00	486.00	.368
Syntax:Expertise	1.15	1.00	486.00	.284
Semantics:English	2.85	1.00	486.00	.092
*Syntax:English	4.11	1.00	486.00	.043

Note. Semantics = semantic plausibility; Syntax = syntactic validity; Expertise = Python expertise; English = English proficiency. Due to the categorical nature of Semantics, Syntax, and Electrode, parameter estimates are unstandardized; however, Expertise and English were z-transformed prior to being entered into the model. The Satterthwaite method was used to estimate degrees of freedom and generate *p*-values. *significant at an alpha of .05.

Supplementary Table S15.
Estimates of fixed effects for the linear mixed model predicting ERP responses in the N400 window, with the inclusion of English proficiency as a predictor.

			95% CI				
**Parameter**	**Estimate**	***SE***	**Lower**	**Upper**	***df***	***t***	***p***
Intercept	-0.02	0.39	-0.79	0.75	42.00	-0.05	.964
*Semantics	-1.71	0.20	-2.10	-1.32	486.00	-8.62	<.001
*Syntax	1.40	0.20	1.01	1.79	486.00	7.05	<.001
Expertise	0.04	0.04	-0.04	0.13	42.00	1.00	.323
English	0.05	0.44	-0.82	0.91	42.00	0.11	.917
Electrode (Fz - Cz)	0.31	0.24	-0.17	0.79	486.00	1.27	.206
*Electrode (Cz - Pz)	-1.18	0.24	-1.65	-0.70	486.00	-4.84	<.001
Semantics:Syntax	0.64	0.40	-0.14	1.41	486.00	1.60	.111
Semantics:Expertise	0.02	0.02	-0.02	0.06	486.00	0.90	.368
Syntax:Expertise	0.02	0.02	-0.02	0.07	486.00	1.07	.284
Semantics:English	-0.38	0.22	-0.82	0.06	486.00	-1.69	.092
*Syntax:English	0.45	0.22	0.02	0.89	486.00	2.03	.043

Note. Semantics = semantic plausibility; Syntax = syntactic validity; Expertise = Python expertise; English = English proficiency; Fz = midline frontal; Cz = midline central; Pz = midline parietal. Due to the categorical nature of Semantics, Syntax, and Electrode, parameter estimates are unstandardized; however, Expertise and English were z-transformed prior to being entered into the model. The Satterthwaite method was used to estimate degrees of freedom and generate *p*-values. *significant at an alpha of .05.

Supplementary Table S16.
Random components for the linear mixed model predicting ERP responses in the N400 window, with the inclusion of English proficiency as a predictor.

**Effect**	**Name**	***SD***	**Variance**	**ICC**
Subject	(Intercept)	2.54	6.45	0.55
Residual		2.31	5.33	

Supplementary Table S17.
Tests of fixed effects for the linear mixed model predicting ERP responses in the P600 window, with the inclusion of English proficiency as a predictor.

**Source**	***F***	**Num *df***	**Den *df***	***p***
Semantics	0.09	1.00	486.00	.761
*Syntax	220.05	1.00	486.00	<.001
*Expertise	4.46	1.00	42.00	.041
English	1.22	1.00	42.00	.275
*Electrode	39.44	2.00	486.00	<.001
Semantics:Syntax	2.93	1.00	486.00	.088
Semantics:Expertise	0.02	1.00	486.00	.875
*Syntax:Expertise	15.77	1.00	486.00	<.001
Semantics:English	0.01	1.00	486.00	.907
Syntax:English	1.86	1.00	486.00	.173

Note. Semantics = semantic plausibility; Syntax = syntactic validity; Expertise = Python expertise; English = English proficiency. Due to the categorical nature of Semantics, Syntax, and Electrode, parameter estimates are unstandardized; however, Expertise and English were z-transformed prior to being entered into the model. The Satterthwaite method was used to estimate degrees of freedom and generate *p*-values. *significant at an alpha of .05.

Supplementary Table S18.
Estimates of fixed effects for the linear mixed model predicting ERP responses in the P600 window, with the inclusion of English proficiency as a predictor.

			95% CI				
**Parameter**	**Estimate**	***SE***	**Lower**	**Upper**	***df***	***t***	***p***
*Intercept	2.07	0.28	1.52	2.62	42.00	7.37	<.001
Semantics	-0.06	0.20	-0.45	0.33	486.00	-0.30	.761
*Syntax	2.97	0.20	2.58	3.36	486.00	14.83	<.001
*Expertise	0.07	0.03	0.005	0.13	42.00	2.11	.041
English	0.35	0.32	-0.27	0.97	42.00	1.11	.275
*Electrode (Cz - Fz)	0.62	0.25	0.14	1.10	486.00	2.54	.011
*Electrode (Pz - Cz)	1.50	0.25	1.01	1.98	486.00	6.10	<.001
Semantics:Syntax	-0.69	0.40	-1.47	0.10	486.00	-1.71	.088
Semantics:Expertise	-0.004	0.02	-0.05	0.04	486.00	-0.16	.875
*Syntax:Expertise	0.09	0.02	0.05	0.14	486.00	3.97	<.001
Semantics:English	0.03	0.23	-0.42	0.47	486.00	0.12	.907
Syntax:English	0.31	0.23	-0.13	0.75	486.00	1.36	.173

Note. Semantics = semantic plausibility; Syntax = syntactic validity; Expertise = Python expertise; English = English proficiency; Fz = midline frontal; Cz = midline central; Pz = midline parietal. Due to the categorical nature of Semantics, Syntax, and Electrode, parameter estimates are unstandardized; however, Expertise and English were z-transformed prior to being entered into the model. The Satterthwaite method was used to estimate degrees of freedom and generate *p*-values. *significant at an alpha of .05.

Supplementary Table S19.
Random components for the linear mixed model predicting ERP responses in the P600 window, with the inclusion of English proficiency as a predictor.

**Effect**	**Name**	***SD***	**Variance**	**ICC**
Subject	(Intercept)	1.76	3.09	0.36
Residual		2.33	5.41	

Supplementary Table S20.
Comparisons of model fit.

Dependent Variable	Model	BIC	R^2 Conditional	R^2 Marginal
Acceptability	Original	1598.46	0.74	0.66
	+ English	1601.75	0.73	0.66
Amplitude (N400 effect)	Original	2631.83	0.60	0.13
	+ English	2659.64	0.61	0.13
Amplitude (P600 effect)	Original	2606.35	0.57	0.32
	+ English	2638.63	0.57	0.33

Note. Acceptability = code acceptability judgments; Amplitude = ERP responses. Original = the models reported in the manuscript; + English = the models that added the effects of English proficiency and its interactions with semantic plausibility and syntactic validity.

Supplementary References

1. Stoet, G. PsyToolkit - A software package for programming psychological experiments using Linux. *Behav Res Methods* **42**, 1096-1104 (2010).
2. Stoet, G. PsyToolkit: a novel web-based method for running online questionnaires and reaction-time experiments. *Teach Psychol* **44**, 24-31 (2017).
